# Welcome to the New Journal *Biomimetics*

**DOI:** 10.3390/biomimetics1010001

**Published:** 2016-02-25

**Authors:** Franck Vazquez

**Affiliations:** MDPI AG, Klybeckstrasse 64, 4057 Basel, Switzerland; biomimetics@mdpi.com

Over geological time and through natural selection, living organisms have evolved specific organs, structures and materials to perform specific functions and allow them to survive and thrive in their environment.

More and more research approaches are driven by aiming at understanding natural biological models at the macro, micro or nano levels, and to imitate these systems to develop products that are useful to humans or to improve the performance of existing ones. Such approaches are called “biomimetics”.

The term “biomimetics” has first been introduced in the 1950s by the American biophysicist, Otto Schmitt, who had already been using biomimetics principles during his doctoral studies to engineer devices mimicking electric signal propagation in squid nerves [1]. Biomimetics principles, although not yet named as such, had been used earlier by others, for example Leonardo da Vinci (1452–1519), who had long been observing and trying to mimick the anatomy and the flight of birds to design his so-called flying machines.

Biomimetics has given rise to new technologies, or great improvements of old technologies,. and can potentially improve applications in all domains of human life, including material sciences, engineering, or medicine.

Famous examples include: (1) Velcro which was invented in 1955 as a novel type of zip fastener mimicking the action of the hooked seeds of the burdock plant; (2) new types of plane wings curved at their distal end to mimic the wings of eagles soaring in the sky to improve plane stability; and (3) a new structure of boat hulls covered in nanostructures mimicking shark skin to improve boat speed and decrease fuel consumption.

The main concept of biomimetics is that nature and evolution have already solved many of the future engineering problems, such as self-assembly, harnessing solar energy, self-healing abilities, and others. No doubt biomimetics will be a central theme for the development of new applications in the near future.

The journal *Biomimetics* (ISSN 2313-7673) aims to be a central forum for researchers engaged in biomimetics research to emphasize their approach to understand the surrounding biological world and their willingness to use its beauty to develop new applications and better products. Let’s wish *Biomimetics* a bright future.
